# How Well Do COP22 Attendees Understand Graphs on Climate Change Health Impacts from the Fifth IPCC Assessment Report?

**DOI:** 10.3390/ijerph15050875

**Published:** 2018-04-27

**Authors:** Helen Fischer, Stefanie Schütte, Anneliese Depoux, Dorothee Amelung, Rainer Sauerborn

**Affiliations:** 1 Department of Psychology, Heidelberg University, 69117 Heidelberg, Germany; dorothee.amelung@psychologie.uni-heidelberg.de; 2 Centre Virchow-Villermé for Public Health, Paris 75004, France; Stefanie.schutte@parisdescartes.fr (S.S.); anneliese.depoux@uspc.fr (A.D.); 3 Institute for Public Health, University Hospital, Heidelberg 69120, Germany; Rainer.sauerborn@urz.uni-heidelberg.de

**Keywords:** IPCC report, Health impacts, understanding of graphs, evidence-based science communication

## Abstract

Graphs are prevalent in the reports of the Intergovernmental Panel on Climate Change (IPCC), often depicting key points and major results. However, the popularity of graphs in the IPCC reports contrasts with a neglect of empirical tests of their understandability. Here we put the understandability of three graphs taken from the Health chapter of the Fifth Assessment Report to an empirical test. We present a pilot study where we evaluate objective understanding (mean accuracy in multiple-choice questions) and subjective understanding (self-assessed confidence in accuracy) in a sample of attendees of the United Nations Climate Change Conference in Marrakesh, 2016 (COP22), and a student sample. Results show a mean objective understanding of *M* = 0.33 for the COP sample, and *M* = 0.38 for the student sample. Subjective and objective understanding were unrelated for the COP22 sample, but associated for the student sample. These results suggest that (i) understandability of the IPCC health chapter graphs is insufficient, and that (ii) particularly COP22 attendees lacked insight into which graphs they did, and which they did not understand. Implications for the construction of graphs to communicate health impacts of climate change to decision-makers are discussed.

## 1. Introduction

The Intergovernmental Panel on Climate Change (IPCC) has arguably the most wide-spun process of assessing the current state of climate change knowledge. As climate change, its impacts on society, and ways to prevent them is a trans-disciplinary effort, IPCC chapters should be scientifically precise, yet also understandable to the expert audience from a wide range of fields to ensure informed decision-making. It is a widespread belief that graphs are both an effective and efficient means for communicating scientific information [1]: Graphs appear well-suited to render complex information easier to understand, to say “more than a thousand words”, and graphs are believed to condense information efficiently to save space. Graphs are also prevalent in the IPCC reports, often depicting key points and major results. However, the popularity of graphs in the IPCC reports contrasts with a neglect of empirical tests of their understandability. In fact, it has been argued before that communicating science requires the systematic feedback of empirical evaluation [2]. Here we put the understandability of three graphs taken from the Health chapter (One of the authors of the present article (RS) was a lead author of an author team of 11 of the health chapter) of the Fifth Assessment Report [3] to an empirical test. Specifically, we evaluate understandability in a sample of attendees of the United Nations Climate Change Conference in Marrakesh, 2016 (COP22), and a student sample by estimating: (i) Objective understanding: How well do recipients understand the messages conveyed by the graphs? (ii) Subjective understanding: How confident are recipients that they understood the message conveyed by the graphs? And (iii) Calibration: How well-aligned is recipients’ subjective confidence that they understood the graph with their actual, objective understanding?

The health chapter summarizes the direct and indirect impacts of climate change on human health and means for adaptation, and seems particularly apt to assess the understandability of the condensed scientific information conveyed in chapter graphs. This is because scientific evidence on climate change and human health has increased considerably since the last assessment report [4], while at the same time, space constraints to communicate this novel evidence were tight: The whole chapter was allocated 30 pages including graphs and tables and 300 references. Chapter authors may therefore feel tempted to compress information into data-rich graphs [5]. 

**Figure 1:**
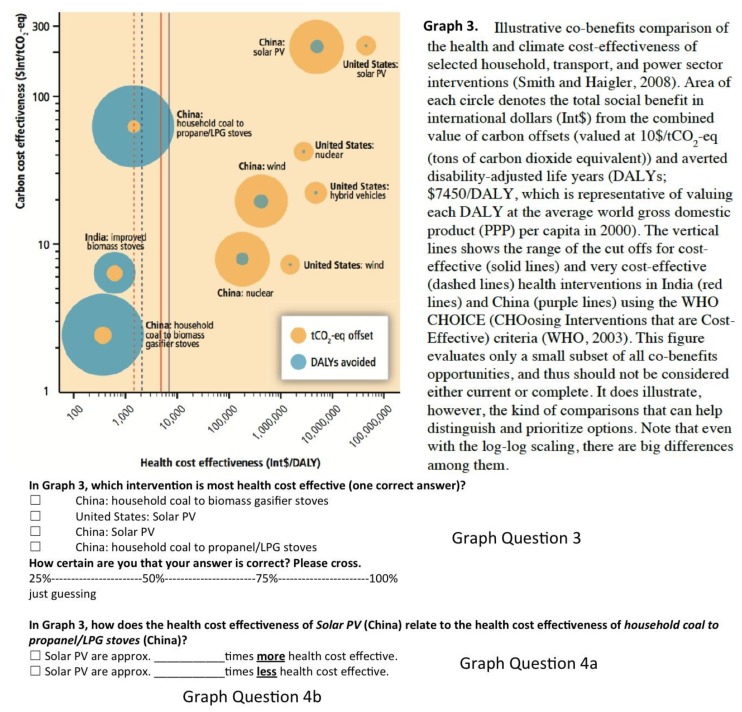
Sample graph taken from the International Panel on Climate Change (IPCC)’s chapter on human health, together with objective and subjective understanding questions. Here: Graph 3, together with Graph questions 3, 4a and 4b.

Two types of characteristics influence how well graphs are understood: top-down characteristics of the recipient viewing the graph, and bottom-up characteristics of the graph itself [6]. Concerning characteristics of the graphs, graphs that display relatively small numbers of variables and data points tend to be easier to understand, because recipients are more likely to be able to attend to all the information displayed. In more complex graphs, in contrast, recipients typically need to select relevant information from a larger amount of information displayed [7]. The health chapter graphs display a relatively large number of variables and data (Figure 1). We therefore expect the graphs to be hard to comprehend. 

Previous research showed that domain-specific knowledge (expertise), ability to reason with numbers (numeracy), and the ability to understand information presented graphically (graph literacy) are relevant characteristics of the recipient that affect graph comprehension. Specifically, expertise is known to facilitate graph comprehension. Expert meteorologists, for example, tend to cluster features from weather maps on the basis of meaningful causal relationships, whereas novices to meteorology cluster the information on the basis of surface similarity [8]. Given that IPCC graphs are typically information-dense, we expect expertise in the area of climate change to facilitate their understanding. High numeracy is known to be less important for the comprehension of relatively simple graphs [9], but facilities the comprehension of more complex graphs [10], and should therefore influence comprehension in the present case of the relatively complex graphs. Graph literacy in turn was found to influence the comprehension of graphs even among highly numerate and well-educated people [11]. We therefore expect participants’ ability to understand graphically presented information to influence graph comprehension above their numeracy skills. 

Recipients of IPCC chapters should not only be able to understand the messages conveyed by the graphs. They should also be aware of those aspects they do not understand. Calibration of understanding refers to how well subjective confidence in one’s understanding aligns with actual understanding. Take two people who answer questions on IPCC graphs. Both of them are 80% confident in the accuracy of their answers. However only one of them, who indeed answered 80% of questions correctly, is well-calibrated. The other one who answered only 40% of questions correctly is overconfident. Crucially, research suggests that lacking insight into the accuracy of one’s judgment can impair the quality of subsequent decision-making. Physicians who are overconfident about their diagnosis being correct tend to prematurely narrow down the choice of diagnostic hypotheses, make more diagnostic error [12], and request fewer additional tests [13]. In the area of political-decision-making, it was found that bureaucrats who are overconfident in their expertise tend to choose more risk-taking policies [14].

Prior research has shown that experts’ calibration—that is, the correspondence of their subjective accuracy with objective accuracy—varies widely. Experts in some areas are very well-calibrated in judgments related to their area of expertise. For example, the subjective and objective accuracy of meteorologists’ predictions are comparatively well-aligned ([15]; see [16], for similar results with bridge players). Other experts tend to be poorly calibrated (for example, physicians: [13]; and lawyers: [17]). What is largely unknown, however, is how well-calibrated expert audiences are in assessing their understanding of scientific climate change information. Here we estimate how well experts’ subjective confidence in their understanding of the graphs aligns with their objective understanding.

Attendees of the United Nations Framework Conference in Marrakesh, 2016 (COP22) answered questions on three graphs from the health chapter. This allowed us to gather data on how relevant audiences understand these graphs. The presented results should be considered pilot since our sample of COP22 attendees constitutes a relevant, but not representative sample of the entire audience of the IPCC reports. A lay-sample without particular interest or expertise in climate change answered the same questions for comparison, namely Heidelberg university mathematics students. They contrasted with the climate experts in that mathematics students (i) likely possess less top-down knowledge on climate change and health; but (ii) are likely to be highly numerate, and more used to reason with complex graphs. This comparison can help estimate the extent to which domain-specific knowledge versus proficiency to reason with information-dense graphs and numbers facilitate understanding of the IPCC health chapter graphs. Furthermore, the comparison can help estimate the degree to which experts compared to novices have an insight into which aspects of the health chapter graphs they do, and which they do not understand.

## 2. Materials and Methods 

### 2.1. Participants 

For the COP22 sample, attendees of the COP22 in Marrakesh, 2016 were asked whether they wanted to fill in a questionnaire on the communication of climate science that would take approximately 10 min. Attendees were approached both in the “green zone” that is accessible to the interested public, and the “blue zone” that requires UN accreditation. A total of *n* = 58 attendees volunteered to participate, without any incentives offered. The sample consists of three groups: (1) Politics, government, and diplomacy; (2) Academia; and (3) other (e.g., NGO, private sector), with participants coming from a total of twenty-three countries. The student sample consisted of a total of *n* = 82 Heidelberg University recruited in a mathematics lecture. See Table 1 for a complete description of the sample composition.

### 2.2. Objective and Subjective Understanding of Health Chapter Graphs

The health chapter contains a total of seven graphs, two of which were newly-developed for the report, and five of which were taken from scientific publications directly. To assess participants’ objective understanding, three graphs from the chapter were selected. The basis for selecting these graphs was (a) to include graphs that depict numerical relations, which the vast majority of graphs do, but which excludes the “conceptual diagram” in the health chapter ([3], p. 717); and (b) to include both a graph that was newly devolved for the chapter ([3], p. 735; here: Graph 1), as well as graphs that were taken from scientific publications ([3], p. 732 and p. 737, here: Graph 2 and Graph 3). The selected graphs can be found in Figure 1 and the Appendix A: Graph 1 displays the heath impacts from climate change and the potential for adaptation; Graph 2 displays the relationship between wet bulb globe temperature, job exertion required, and work output; and Graph 3 displays health and climate cost effectiveness of selected interventions for different countries. 

A total of five graph questions were asked. All questions were chosen to be “simple tasks” in the sense of Canham and Hegarty [7] in that they involve reading off values from graphs, as opposed to inferring new information. Moreover, questions were chosen such that they capture core information depicted in the graphs. Readability of the graphs was assured. Specifically, each graph and respective questions was presented on one separate A4-paper so that each graph covered approximately half a page and all details were clearly visible. We asked one multiple-choice question with four answer options for each graph (Graph Questions 1–3). Specifically, in Graph 1 we asked which climate option bears the greatest potential for risk reduction, in Graph 2 we asked about the specific relationship between temperature and work productivity, and in Graph 3 we asked which intervention is the most health cost effective. In Graph 3, an additional question was asked that consisted of both a multiple-choice part with two answer options (Graph Question 4a), and one open question part where participants needed to give a numerical value (Graph Question 4b). That question was specifically tailored to assess recipients’ understanding of two potentially critical aspects of the x-axis displaying health cost effectiveness: (a) the x-axis is inverted in that health cost effectiveness decreases from left to right; and (b) the x-axis uses a logarithmic scale that has been used previously to visualize comparative respective risks, yielding mixed results [10,18]. Both aspects go against the strong human intuition of increasing linearity. To assesses both critical aspects, we asked (a) which of two measures is more health cost effective, and (b) by how much. 

“Subjective understanding” was assessed for multiple-choice questions 1–3. After answering each question, participants indicated their confidence that the answer they just gave was accurate: “How certain are you that your answer is correct?”, where 25%: just guessing, up to 100%: completely certain in increases of one-percentage point. 

### 2.3. Numeracy and Graph Literacy

To assess numeracy and graph literacy, two numeracy items were taken from [19] and two graph literacy items were taken from [20,21]. Only four items were carefully selected based on this previous research due to time constraints for conducting the survey during the COP conference. For illustration of a sample item, one of the numeracy items was: “The chance of getting a viral infection is 0.0005. Out of 10,000 people, about how many of them are expected to get infected?”, see Appendix A (Figure A2) for all numeracy and graph literacy items.

### 2.4. Dependent Variables

*Objective understanding* was measured as each participant’s accuracy in answering a graph question. Mean accuracy hence measured each participant’s mean accuracy to all graph questions. *Subjective understanding* was measured as each participant’s confidence that they answered a question correctly. To measure calibration, the C-Index was used. The C-Index determines how much subjective confidence in understanding diverges from objective understanding by assessing the mean weighted difference between subjective confidence and objective understanding, computed for each level of confidence: 1/N·Σ n(r−c)^2^. N is the total number of tasks where subjective confidence ratings were elicited, n is the number of probabilities for each category, r is the numerical value of probability for category, c is the proportion of correct answers in each category [22]. To do so, participants’ continuous confidence ratings were collapsed in three confidence categories: 25–50%, 51–75%, and 76–100% ([22]; 50% and 75% were attached to the lower of two adjacent categories each to reduce overconfidence). To measure over-/underconfidence, the same formula as for calibration was used, only that the differences were not squared to achieve directed deviations: Positive values denote for overconfidence, negative values underconfidence.

### 2.5. Procedure

Participants answered the questions assessing objective and subjective understanding of the graphs, followed by the two numeracy and the two graph literacy items. Lastly, participants answered a range of person characteristics and demographics, namely two climate change belief questions (“How much do you think climate change can influence health?” and “How much do you think climate change is caused by humans?”; where 0: not at all, up to 10: very much), sex, age, nationality, education, field of education, political attitude (“How would you characterize your political attitude?”; 5-point scale left–right), and employment.

## 3. Results

### 3.1. Objective and Subjective Understanding by Graph

Mean accuracy did not vary between the COP22 (*M* = 0.33, *SD* = 0.19) and the student sample (*M* = 0.38, *SD* = 0.21), *t*(138) = −1.6, *p* = 0.12. Descriptive results of subjective and objective understanding per graph are given in Table 2, separate for the COP22, and the student sample. Since questions 4a and 4b test two aspects of one and the same graph feature (x-axis of Graph 3 displaying health cost effectiveness, Figure 1), we additionally give the mean results of whether both aspects were answered correctly. Please note for the numerical question 4b, 10,000 would be the correct answer, but also 1000 was counted as correct to account for rounding off values at the x-axis.

### 3.2. Numeracy and Graph Literacy

Mean numeracy was lower for the COP22 (*M* = 0.48, *SD* = 0.40) than the student sample (*M* = 0.84, *SD* = 0.29), *t*(75.6) = −5.15, *p* < 0.001. Graph literacy, however, did not differ between the COP22 (*M* = 0.38, *SD* = 0.37) and the student sample (*M* = 0.47, *SD* = 0.30), *t*(100.3) = −1.31, *p* = 0.20. Numeracy was correlated to mean accuracy for the COP22 sample, *r*(44) = 0.36, *p* = 0.016, but unrelated for the student sample, *r*(55) = −0.15, *p* = 0.29. Graph literacy was unrelated to mean accuracy in both the COP22, *r*(55) = −0.05, *p* = 0.70, and the student sample, *r*(48) = 0.05, *p* = 0.75. 

### 3.3. Calibration and Over-/Underconfidence

As is typically found, overconfidence was strongly and negatively associated with accuracy for both the COP22, *r*(57) = − 0.66, *p* < 0.001, and the student sample, *r*(81) = −0.53, *p* < 0.001, suggesting that overconfidence in one’s understanding increased as actual understanding decreased. Mean calibration (C-Index) did not differ between the COP22 (*M* = 0.17, *SD* = 0.16) and the student sample (*M* = 0.14, *SD* = 0.14), *t*(138) = 1.4, *p* = 0.16. The signed over-/underconfidence, however, was higher for the COP22 (*M* = 0.14, *SD* = 0.29) than the student sample (*M* = 0.04, *SD* = 0.25), *t*(138) = 1.99, *p* = 0.048. Moreover, subjective and objective understanding were unrelated for the COP22 sample, *r*(57) = 0.10, *p* = 0.45, but associated for the student sample, *r*(82) = 0.29, *p* = 0.009.

## 4. Discussion

Decisions in the context of climate change and health need to be based on the best scientific evidence available in order to be most effective. Since the human health impacts of climate change generate a large and increasing number of scientific publications per year, however, it would be close to impossible to keep track. The IPCC regularly provides a unified scientific signal to communicate policy-relevant evidence. Here we assessed objective and subjective understanding of health chapter graphs in attendees of the COP22 and a sample of mathematics students. Evidence on how these graphs are interpreted seems necessary, both because large variation was found in how decision-makers interpret climate data in previous research [23], and because appropriate development of data visualizations is fundamental to guide adaptation decisions.

**Figure A1:**
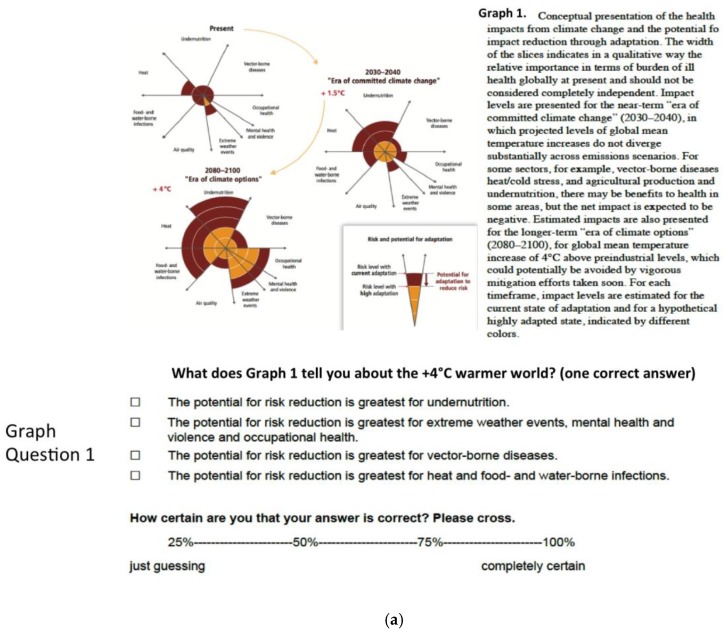

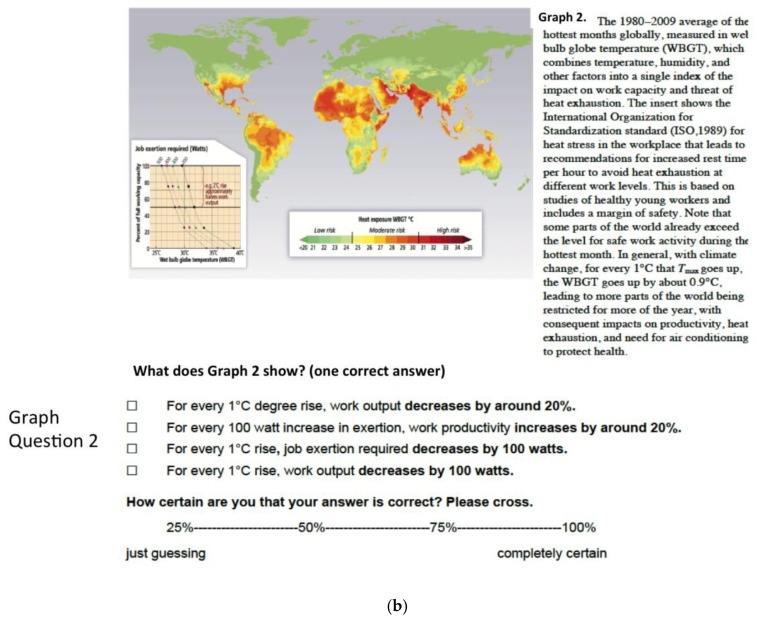
Health chapter graphs and questions. (**a**) Graph 1 together with graph question 1. (**b**) Graph 2 together with graph question 2.

**Figure A2:**
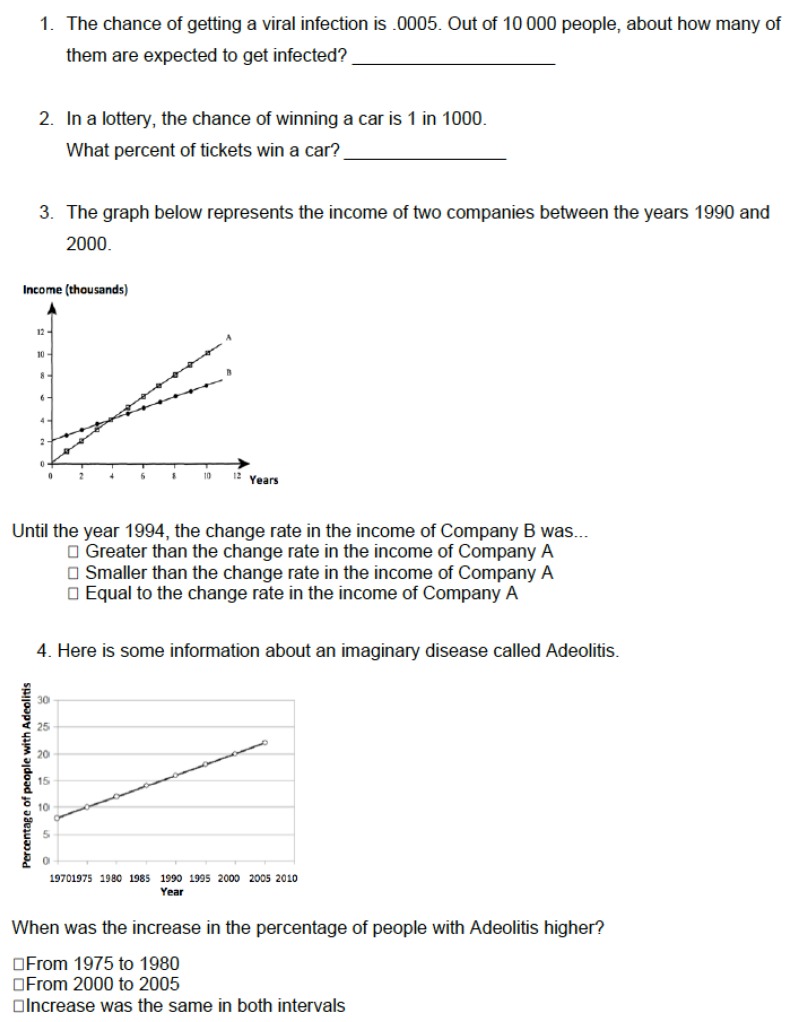
Numeracy and graph literacy items.

With approximately 50% accuracy each, the COP22 sample could best understand Graph 1 (see Figure A1) depicting health impacts of climate change that was newly developed for the health chapter, and Graph 2 (see Figure A1) depicting the rather intuitive relationship between temperature and work output. The COP22 sample had the most difficulties understanding Graph 3 depicting health cost effectiveness of selected interventions. This graph’s x-axis displaying health cost effectiveness seemed to pose a particular barrier to understanding. Specifically, 22% of the sample were able to pinpoint the most health cost effective intervention, 26% were able to indicate which of two given measures is more health cost effective, and only 15% could read off by how much health cost effectiveness increases from one measure to another. 

Results on the low understandability of Graph 3, particularly the scaling of its x-axis, are interesting for two reasons. First, they underline the importance of finding suitable and easy-to-understand means of communicating key findings. It seems hardly satisfying when only one quarter of recipients correctly reads off the main message meant to be conveyed by the graph. Second, the consistent pattern of errors that recipients made is revealing. When asked which of two given measures is more health cost effective, approximately three quarters of the sample (incorrectly) picked the one displayed to the right along the x-axis, rather than (correctly) picking the one to the left. This consistent mistake probably reflects recipients’ intuition that quantities increase from left to right, rather than from right to left. Similarly, when asked to indicate the factor by which health cost effectiveness increases or decreases from one of the measures to the other, particularly the COP22 sample (to a lesser degree: the student sample) seemed to be confused by the logarithmic scale. The most common answer was “2”, which would align approximately with the distance between those measures if the scale were linear (Figure 1). These typical mistakes suggest that recipients had the too strong expectation of a linear increase from left to right when trying to make sense of the graph. It therefore seems advisable to design graphs in a way that exploits these expectations, rather than rely on overcoming them. 

Although the present study provides potentially useful first results on the understandability of the IPCC health chapter graphs, some limitations of this study must be acknowledged and discussed. First, our sample of COP22 attendees does not constitute a representative sample of either the primary target audience (i.e., governments and policy-makers) or broader audiences (i.e., the scientific community, non-governmental organizations, the business sector, or the wider public). Although all of these groups were represented in the COP22 sample, it remains unclear whether the present results hold for representative samples for each of these audiences. For the very common mistake of assuming linearity, however, the present results might arguably not be subject to much variation since previous research has shown that linearity constitutes a very fundamental human intuition [24]. Second, we did not track the number of people who were asked whether they wanted to take part during the conference, and therefore cannot give the response rate. Since people were approached by asking whether they wanted to take part in a study on understandability of IPCC graphs, it seems likely that though a self-selection effect our participants were interested in this topic. Future studies should therefore aim for representative samples of relevant audiences to estimate understanding of the graphs. Third, we did not incentivize correct answers which might undermine participants’ motivation to engage with the graphs. Although accuracy of understanding might not necessarily be incentivized in real-world situations, future studies should investigate the extent to which graph comprehension changes when accuracy is incentivized. In sum, the present pilot study does not allow to draw conclusions on the understandability of IPCC health graphs for a representative sample of the IPCC audience. It does, however, show the need to study understandability of IPCC graphs more in-depth in future studies. 

Concerning characteristics of the respondents that influenced graph comprehension, we found a substantial association with numeracy (but not graph literacy) in the COP22 sample, in that more numerate attendees were better able to understand the graphs. This result underlines the rather scientific style of the health chapter graphs that make ample use of mathematical concepts such as geometry of circles (Graph 1), non-linear relationships, displayed on more than two axes (Graph 2), or logarithmic scaling (Graph 3). It is important to note that the use of complex, numerical elements is not specific to those graphs that were selected for the present research, but appears rather typical of the health chapter. The present results suggest that relying on strong numerical skills less could be an effective means to increase understanding of the key messages conveyed by the health chapter graphs.

Objective understanding was comparable for the COP22 and the student sample. Interestingly, though, the COP22 sample more strongly overestimated their actual understanding compared to the students. That is, COP22 attendees were more confident that they understood the graphs than justified by their actual understanding. Similarly, subjective confidence in understanding was associated with objective understanding for the student, but not the COP22 sample. These results suggest that COP22 attendees tended to have a feeling-of-understanding, even if they did not in fact understand the graphs, and that they lacked insight into which health chapter graphs they did, and which they did not understand.

## 5. Conclusions

In COP22 attendees, objective understanding of graphs taken from the health chapter of the latest IPCC report (IPCC, 2014) varied between 14% and 50% per question. This result seems particularly worrisome because recipients, to a large degree, had no insight into their lack of understanding. Results showed that participants made particularly consistent mistakes when the graphs contradicted intuitive assumptions, such as that scales should increase linearly from left to right. By making use of results on design features of health graphs [25], understandability of these graphs might be improved. 

## Figures and Tables

**Table 1:** United Nations Climate Change Conference in Marrakesh, 2016 (COP22) and student sample composition.

Variable	COP22 Sample		Student Sample	
	Value	Min.–Max.	Value	Min.–Max.
Age	Mean: 36 (11.2)	21–75	Mean: 21.8 (3.5)	14–30
Female	49%		22%	
Country (Frequencies)	Belgium (2), Brazil, China (2), Denmark, EU, France (4), Germany (7), Grenada, Guyana, India, Italy (4), Korea, Malta, Mexico, Morocco (17), Portugal, Romania, Spain (2), Sweden (2), Thailand, Turkey, USA, Yemen		Germany (35), Austria, China, India, New Zealand, Pakistan, Turkey	
Education	HighSchool: 3 (6%) Bachelor: 10 (18%) Master: 26 (48%) PhD: 15 (28%)		HighSchool: 20 (40%) Bachelor: 17 (33%) Master: 11 (22%) PhD: 3 (6%) No answer: 31	
Employment	Politics, Government, Diplomacy: 15 (29%) Academia: 14 (27%) Other (NGO, private sector, press, consulting): 23 (44%)		-	

**Table 2:** Objective and subjective understanding of the health chapter graphs in the COP22 and the student sample.

Graph Question	COP22 Sample			Student Sample		
	Objective Understanding Mean (SD)	Most Frequent Answer Option/Answer	Subjective Understanding Mean (SD)	Objective Understanding Mean (SD)	Most Frequent Answer Option/Answer	Subjective Understanding Mean (SD)
1	0.50 (0.50)	#1 (correct answer)	0.66 (0.24)	0.77 (0.42)	#1 (correct answer)	0.68 (0.23)
2	0.51 (0.50)	#1 (correct answer)	0.53 (0.24)	0.50 (0.50)	#1 (correct answer)	0.57 (0.24)
3	0.22 (0.42)	#2 (incorrect answer)	0.63 (0.25)	0.26 (0.44)	#2 (incorrect answer)	0.62 (0.25)
4a	0.26 (0.44)	#1 (incorrect answer)	-	0.15 (0.36)	#1 (incorrect answer)	-
4b	0.14 (0.35)	2	-	0.24 (0.43)	10,000	-
4a and 4b	0.02 (0.13)		-	0.06 (0.24)		-
